# Predicting Metabolic and Cardiovascular Healthy from Nutritional Patterns and Psychological State Among Overweight and Obese Young Adults: A Neural Network Approach

**DOI:** 10.3390/nu17162651

**Published:** 2025-08-15

**Authors:** Geovanny Genaro Reivan Ortiz, Laura Maraver-Capdevila, Roser Granero

**Affiliations:** 1 Faculty of Clinical Psychology, Catholic University of Cuenca, Cuenca 010107, Ecuador; greivano@ucacue.edu.ec; 2 Department of Psychobiology and Methodology, Universitat Autònoma de Barcelona, 08193 Barcelona, Spain; laura.maraver@uab.cat

**Keywords:** overweight, obesity, nutrient patterns, cardiometabolic, body mass index, HOMA-IR

## Abstract

Background and objectives: Overweight and obesity are global public health problems, as they increase the risk of chronic diseases, reduce quality of life, and generate a significant economic and healthcare burden. This study evaluates the capacity of nutritional patterns and psychological status to predict the presence of cardiometabolic risk among overweight and obese young adults, from a neural network approach. Method: The study included *N* = 188 overweight or obese students, who provided measures on their dietary intake, physical and psychological state, and sociodemographic profile. Neural networks were used to predict their metabolic status, classified into two categories based on anthropometric, biochemical, and cardiometabolic risk factors: metabolically unhealthy obesity (MUO) versus metabolically healthy obesity (MHO). Results: The predictive models demonstrated differences in specificity and sensitivity capacity depending on the criteria employed for the classification of MUO/MHO and gender. Among the female subsample, MUO was predicted by poor diet (low consumption of mineral and vitamins, and high consumption of fats and sodium) and high levels of depression and stress, while among the male subsample high body mass index (BMI), depression, and anxiety were the key factors. Protective factors associated to MHO were lower BMI, lower psychopathology distress and more balanced diets. Predictive models based on the HOMA-IR criterion yielded very high specificity and low sensibility (high capacity to identify MHO but low accuracy to identify MUO). The models based on the IDF criterion achieved excellent discriminative capacity for men (specificity and sensitivity around 92.5%), while the model for women obtained excellent sensitivity and low specificity. Conclusions: The results provide empirical support for personalized prevention and treatment programs, accounting for individual differences with the aim of promoting healthy habits among young adults, especially during university education.

## 1. Introduction

Overweight and obesity among young adults are increasingly prevalent conditions worldwide and are associated with significant risks to both physical and psychological health [1,2]. They are primarily defined by body mass index (BMI), a standard measure to assess the amount of body fat. According to the World Health Organization, overweight is defined as a BMI between 25 and 29.9 kg/m^2^, while obesity is a BMI exceeding 30 kg/m^2^ [3]. Each year, approximately 2.8 million people die worldwide due to clinical conditions related to the presence of overweight and obesity. The causes of these morbidities are multiple and complex, involving a combination of nutritional, psychological, physical, and social factors [4].

Dietary patterns play a fundamental role in the onset of overweight and obesity [5]. Excessive intake of ultra-processed foods, which tend to be high in empty calories, saturated fats, and sugars, along with a lack of nutrient-rich foods such as fruit, vegetables, and lean proteins, contribute significantly to weight gain [6]. In parallel, a sedentary lifestyle, common in many young adults due to the time spent in front of screens and labour or academic activities that require little physical activity, is also a crucial factor [7]. A lack of regular exercise makes weight control difficult, as calorie expenditure is insufficient to offset calorie consumption [8]. Psychological factors such as stress, anxiety and depression also influence eating habits, as many people turn to food to cope with negative emotions, which can trigger episodes of overeating and hence further weight gain [9,10].

Studies have observed that the proportion of overweight or obese individuals increases with age [11], particularly from midlife (age 50–64) to early late-life (age 65–79) [12,13,14,15]. These studies suggest that increased BMI is strongly related to the physiological changes [16] associated with the aging process, including metabolic decline [17,18]. However, the rapid changes in lifestyle in recent decades (including eating habits and reduction of physical activity) has contributed to a growing prevalence of obesity and overweight worldwide [19] among all age groups [20], with empirical findings suggesting that about 70–80% of obese adolescents will remain obese in adulthood. These studies have noted that socioeconomic and cultural factors also play a significant role in the prevalence of overweight and obesity in young adults [21,22]. Limited access to healthy food and the influence of certain cultural and social norms can influence dietary choices and physical activity levels [23,24]. These conditions not only have immediate repercussions in terms of health but can also lead to long-term complications [25].

Obesity can be classified as metabolically healthy or metabolically unhealthy [26,27]. Based on classic models of metabolic and cardiovascular risk, metabolically healthy obesity (MHO) refers to individuals with excess body fat but no significant metabolic abnormalities, such as insulin resistance, dyslipidaemia, hypertension, chronic inflammation, and diabetes [28,29]. Conversely, patients with excess body fat and which do present these risks are known as metabolically unhealthy obese (MUO). Studies have highlighted that this latter type of obesity is linked to the accumulation of visceral fat, which negatively impacts metabolism and can lead to long-term complications, even in young people without an overtly obese physical appearance [30,31]. In this regard, the HOMA-IR (Homeostasis Model Assessment of Insulin Resistance) and IDF (International Diabetes Federation) classifications are useful instruments for assessing metabolic syndrome and obesity-associated risks [32,33]. HOMA-IR measures insulin resistance, a key factor in the onset of type 2 diabetes and cardiovascular disease. A high HOMA-IR value indicates metabolic dysfunction, even in the absence of obvious diabetic symptoms, and helps identify high-risk individuals [34,35]. The IDF is used to diagnose metabolic syndrome, which is defined as a combination of risk factors such as abdominal obesity, hypertension, and high glucose and triglyceride levels [32,36]. It has been observed that early detection of this metabolic syndrome can help to reduce the risk of developing long-term chronic disease [37].

The distinction between MHO and MUO individuals has gained increasing attention in recent years, given its implications for understanding the heterogeneity of obesity-related health risks. At present, HOMA-IR and IDF stand out as robust indicators for assessing metabolic health in individuals with obesity and constitute the most widely used and validated approaches [38]. However, controversies persist. For example, individuals within the normal HOMA-IR group but lacking metabolic syndrome components are often categorized as MHO despite persistent adiposity-associated dysregulation [39]. Early identification of risk factors for cardiometabolic complications involving young adults is critical for timely prevention plans. And because the metabolic related problems can remain clinically silent during youth, relying on a single diagnostic criterion may underestimate the true burden of risk. The combination of two empirical-based classification systems (IDF and the HOMA-IR criteria, developed for capturing a broad constellation of metabolic syndrome components), provides a more comprehensive assessment of metabolic health. The use of both criteria increases the likelihood of detecting subclinical alterations and enhances the predictive validity of early markers associated with (severe) (long-term) cardiometabolic outcomes.

On the other hand, research has highlighted notable differences between the dietary and metabolic patterns of MUO men and women [26,28,38,39,40,41]. Men tend to consume diets rich in refined carbohydrates and saturated fats, which promote insulin resistance, dyslipidemia, and metabolic disorders, in addition to having a higher sodium intake, which contributes to hypertension. Meanwhile, studies have reported that women, despite consuming more foods rich in fiber and vitamins, face a greater risk of abdominal obesity with age due to deficiencies in minerals such as magnesium and vitamin D [28]. As women age, their insulin sensitivity decreases, which can cause the accumulation of visceral fat [26]. Regarding psychology, research has reported that both men and women with MUO have a higher prevalence of disorders such as anxiety and depression, which are aggravated by stress and the intake of ultra-processed foods [42,43]. Men are particularly prone to emotional consumption of high-calorie foods [44]. Physically, they tend to present greater insulin resistance and elevated levels of glucose, triglyceride, and LDL cholesterol. Despite their more favorable lipid profile, women experience similar changes in glucose and insulin levels after menopause. Therefore, both sexes, albeit at different times, face a higher risk of disorders such as type 2 diabetes and cardiovascular disease as a result of altered metabolic profiles [45].

Most epidemiological and clinical studies aimed at identifying the risk factors and underlying etiological processes of obesity are based on classical statistical procedures, including Generalized Linear Models (GLM), such as linear regression for continuous outcomes, logistic regression for binary responses and Poisson regression for count data. However, recent studies have used a machine learning extension of GLM that employs neural networks to improve the prediction of complex problems in medicine [46] and mental health [47]. This promising tool uses a training algorithm that artificially imitates the human nervous system [48] to iteratively estimate and change the parameters of the internal architecture of the network, including the layout of the system and the processing units, as well as the strength of the connections between them. Neural networks have the advantage of high flexibility and learning capacity for multifaceted non-linear datasets without explicit assumptions about the relationships between variables. Neural networks have been considered less transparent and harder to interpret than classical GLMs, but tend to outperform them for predictive accuracy, especially at the individual level [49]. Due to their excellent capacity for generalized diagnosis and prognosis, these techniques are especially interesting for obesity research, where they can be used to support continuous monitoring of metabolic and cardiovascular status, ultimately promoting better physical and mental well-being.

### Justification and Objectives

Given the early onset and silent progression of metabolic related problems, there is a pressing need for empirical studies aimed at identifying risk factors among overweight and obese young individuals from the general population. The objectives of the present study were to explore the predictive role of both nutritional patterns and psychological variables (depression, anxiety and stress levels) on the binary classification within MHO/MUO based on neural network methodology. This multidimensional approach is intended to improve our understanding of how lifestyle and psychosocial factors contribute to the development of metabolic dysregulation during youth, and to support the design of targeted prevention and intervention strategies.

Based on the accumulated evidence from previous research, we hypothesize that the metabolic status of overweight and obese young individuals is associated with dietary patterns, physical variables, and psychological factors, and that the strength of these associations will differ depending on the operational definition of cardiovascular health.

## 2. Materials and Methods

### 2.1. Participants

The data analysed in this study was recruited as part of a broader research project that examined multiple factors contributing to the cardiometabolic health status of young university students with overweight or obesity. The study included 188 overweight and obese students from the Catholic University of Cuenca, in Azogues, Ecuador, who were recruited by means of intentional volunteer sampling. All participants signed an informed consent form. Individuals were excluded if they were pregnant, had endocrine or genetic disorders (such as hypothyroidism, type 1 diabetes, and Cushing’s syndrome), were on a weight-loss diet, or were taking supplements or medications that could affect glucose or lipid levels, body weight, or blood pressure. The study did not exclude individuals with eating disorders, as its objective was to obtain empirical evidence on the general population of young people with overweight or obesity. This included both individuals with and without eating disorders, thus enhancing external validity and enabling the results to be generalized to a broader and more representative population. The complete description of the sampling method and the sample is available in the work currently published by Reivan and colleagues [50].

### 2.2. Measures

*Consumption Routine.* The Food Frequency Questionnaire (FFQ) [51] was used to record the participants’ diet. A trained nutritionist showed them how to use the tool, asking them to record the frequency (daily, weekly, and monthly) and quantity of foods consumed over the past 12 months, based on standard portion sizes. Total energy and nutrient intake was calculated by summing the values for all foods consumed, using Nutrimind software (https://www.nutrimind.net/, accessed on 25 September 2024) for data processing. In this work we analysed 3 nutritional empirical factors, thoroughly described in the study by Reivan et al. [50]. Briefly, factor analysis was used to extract the three patterns according to the responses of food consumption in the last 12 months. NP1 was the label employed for the factor measuring the high content of minerals and vitamins (potassium, magnesium, folate, pantothenic, acid, riboflavin, phosphorus, zinc, calcium, fibre, and vitamins B12, B6 and C), NP2 for high carbohydrate (thiamine, niacin, carbohydrates and iron), and NP3 for high fat and sodium (monounsaturated, polyunsaturated and saturated fatty acids, and sodium).

*Cardiometabolic Health Factors.* Assessment of cardiometabolic factors and anthropometric indices was performed by a nurse specialized in nutrition. Weight was measured using a calibrated electronic scale (accuracy of 0.1 kg), and height was recorded using a stadiometer (accuracy of 0.1 cm). Waist circumference (WC), a complementary indicator of overweight and obesity [52] was measured twice, and the average of both measurements was used. After a 5-min rest, two blood pressure measurements (diastolic and systolic) were taken 15 min apart, and the average of both measurements was recorded [53]. Biochemical values were obtained through venous blood samples after a 12-h fast, determining glucose, lipid profile, and insulin concentrations. Insulin resistance was calculated using the HOMA-IR model. Two methods were used to classify the participants’ metabolic health status. The first, based on the modified International Diabetes Federation criteria, classifies obesity as metabolically unhealthy if at least two of the following factors are present: elevated triglycerides, high fasting blood glucose, low HDL-C, and high blood pressure. The second method used the HOMA-IR index, based on the fasting glucose and insulin levels and calculated by using the following formula:

HOMA-IR = (fasting glucose [mg/dL] × fasting insulin [mU/L])/405

Alternatively, the formula when glucose is expressed in mmol/L is:

HOMA-IR = (fasting glucose [mmol/L] × fasting insulin [mU/L]/22.5.

Based on the HOMA-IR index, scores greater than 3 indicates MUO, and a scores below 3 indicates MHO [54].

*Anthropometry.* Body Mass Index (BMI) was the primary tool for assessing nutritional status, obtained by dividing weight in kilograms by height in meters squared. The World Health Organization [3] classifies BMI into two categories: overweight (25 ≤ BMI < 30) and obesity (BMI ≥ 30).

*Psychological status.* The Depression, Anxiety, and Stress Scale (DASS-21) [55] was used to assess psychological status. This self-report questionnaire consists of 21 questions about activities in the previous week, with answers on a Likert scale ranging from 0 (never) to 3 (almost always). It also provides a total score reflecting general psychological distress. The internal consistency of the questionnaire was excellent in this study, with α values of 0.80 for depression, 0.82 for anxiety, 0.86 for stress, and 0.90 for the total scale.

*Socioeconomic factors.* In addition to nutritional variables, data were collected on participants’ age, sex, marital status, and socioeconomic status (SES), the latter determined using the Hollingshead Four-Factor Index [56].

### 2.3. Procedure

Participants were recruited with the authorization of the governing authority at the Catholic University of Cuenca and the Program Supervisors. An invitation letter was sent to the students via the institutional email. Those who accepted participated in two assessment sessions. The first session, held in a classroom, was led by two clinical psychologists and a nutritionist trained in the measurement tools. Participants completed questionnaires collecting nutritional, psychological, and sociodemographic data, and the session lasted approximately 40 min. In the second session, participants were scheduled for a specific date and time. A nurse and a clinical psychologist with over 10 years of experience recorded anthropometric and biochemical data. All data were collected in October 2023. Participation was voluntary, with no financial or academic incentives.

### 2.4. Neural Networks

Neural network model is a subset of machine learning based on layered computational architectures, formed by three types of layers [57]: (a) an input layer, where the input-independent variables are fed; (b) one or more hidden layers; and (c) an output layer, that generates predictions or classifications based on the learned representations. Each layer is formed by units called “neurons” that are connected to neurons in adjacent layers through weighted links (the internal structure of neural networks is based on a complex matrix of learned weights). The model propagates information forward through the hidden layers and activation functions transform the weighted sums of inputs allowing the network “to learn” the patterns in the data. The training of the model is based on the re-adjusting of the connection weights according to optimization algorithms aimed at minimizing prediction errors [58].

In the context of neural networks and supervised machine learning, the dataset is typically divided into two distinct separated subsamples, that serves a specific role in the model development and evaluation process: training and testing. The training dataset is the proportion of the sample used to fit the model, employed in the first step for teaching the neural network about the relationships between input features and the target output. This step provides the model the knowledge of the patterns based on the optimization of the internal parameters (weights and biases) through the propagation process. The testing dataset is employed for measuring the model’s performance during the previous training process, based on the capacity of the neural network for generalizing learning to new-unseen data. During the second step, the testing sample is not employed for re-adjusting the model’s parameters, but is often used for hyperparameter tuning or early stopping. The comparison between the model’s predictions with the real outcomes in the two datasets allows the evaluation generalization capacity of the neural network.

In psychiatric research, these techniques are helpful for dealing with multivariate and high-dimensional datasets, allowing modelling where traditional linear approaches may be inadequate [46]: neural networks have the capacity to capture complex relationships in the data, increasing predictive accuracy and enabling the discovery of latent structures. In clinical practice, varios applications based in neural networks methodology are still in the proposal stage, but are considered promising tools in early screening, diagnosis and digital interventions. Some of these applications include mental health chatbots, systems providing feedback on psychotherapy or peer support sessions, and (self-)diagnosis tools that take symptom information from individuals (patients, but also individuals from the general population), process that data, and generate diagnoses (or tailored healthcare advice) and treatment plans [59,60].

### 2.5. Statistical Analysis

Predictive models were obtained using neural networks in SPSS29 for Windows. This supervised learning technique is implemented via the Multilayer Perceptron (MLP) procedure, which is currently the most widely-used prediction method in mental health research. In this work, 75% of the sample was used as the training set and the remaining 25% formed the testing set. The Hyperbolic tangent was defined as the activation function in the hidden layers, and the Softmax activation function was defined in the output layer. Automatic architecture was also specified, and the starting point for the model was set at 20250304 via the “SET SEED” command.

Four separate neural network models were created, one each for women and men, and also for the two criteria employed to identify MUO (HOMA-IR and IDF). The dependent variable was metabolic state (1 = MUO vs. 0 = MHO), and the independent variables (predictors) included age, BMI, psychopathological state (depression, anxiety and stress levels), and the nutritional pattern factor scores (NP1, NP2 and NP3).

The validity of the neural predictive models for discriminating between MUO/MHO was assessed by comparing the models’ predictions with the observed metabolic state. Sensitivity (Se) was calculated as the ability of the MLP model to correctly identify participants with MUO (presence of “disease”), and Specificity (Sp) measured the ability to correctly identify participants with MHO (absence of “disease”). Predictive values were also obtained. For a positive result (calculated as the probability of participants classified as positive by the MLP model actually being cases of MUO) and for a negative result (calculated as the probability of participants classified as negative by the MLP model actually having MHO). Additional indices included the false positive rate (FPR, calculated as the proportion of individuals with MHO that were misclassified by the MLP) and the false discovery rate (FDR, calculated as the proportion of individuals classified as MUO by the MLP whose real state was MHO). The discriminative capacity of the neural network was also assessed with Receiver operating characteristic (ROC) curves and the corresponding Area Under the Curve (AUC values). The ROC curve plots in the x-axis the true positive rate (sensitivity) and the false positive rate (1-specificity) in the y-axis, across the decision thresholds (predictions) generated in the neural network. This plot provides a graphical representation of the model’s capacity to correctly classify the individuals defining as a criterion (the reference gold-standard) the observed clinical state (MUO/MHO), equivalent to how the classification ability of a screening or diagnostic test would be assessed in the medical field [61]. The AUC is a single value measuring the model validity, with values ranging from 0.5 (indicating null discriminative capacity, equivalent to random classification) to 1 (indicating perfect discriminative capacity). The interpretation of the *AUC* for assessing the capacity of binary classifiers (such as neural networks, but also other models like logistic regression) is interpreted as low-poor for *AUC* < 0.60, moderate-median for 0.60 < *AUC* < 0.75, high-large for 0.75 < *AUC* < 0.90 and excellent for *AUC* > 0.90 [62].

## 3. Results

### 3.1. Characteristics of the Sample

The descriptive for the total sample, and stratified by sex, is shown in Table 1. Most participants were single (78.7%, versus 18.6% married and 2.7% divorced). The distribution of the social position index was as follows: 37.8% low, 33.0% within mean and 29.3% high. Mean age was around 21 years (SD = 2.6) and mean BMI was 28.4 kg/m^2^ (SD = 2.9).

Quantitative variables were compared between women versus men using independent samples *t*-Tests (the assumption of normality was considered met, as the large sample size ensures that the sampling distribution of the difference in means approximates a normal distribution in accordance with the Central Limit Theorem). For categorical variables, comparisons were conducted using the chi-square (χ^2^) test to assess differences in proportions. Statistical differences between sexes were obtained for the mean scores in nutritional pattern NP1 (minerals and vitamins), with higher means among men, and for depression and anxiety (worse among women).

### 3.2. Neural Network Model for Women Predicting the Presence of MUO (HOMA-IR Criterion)

Figures S1–S4 (Supplementary Material) show the neural diagrams obtained for the predictive models, stratified by sex and metabolic classification criteria. Grey lines represent synaptic weights higher than 0 (activation), and blue lines represent synaptic weights lower than 0 (inhibition). The parameter estimates are shown in Table 2.

**Figure 1:**
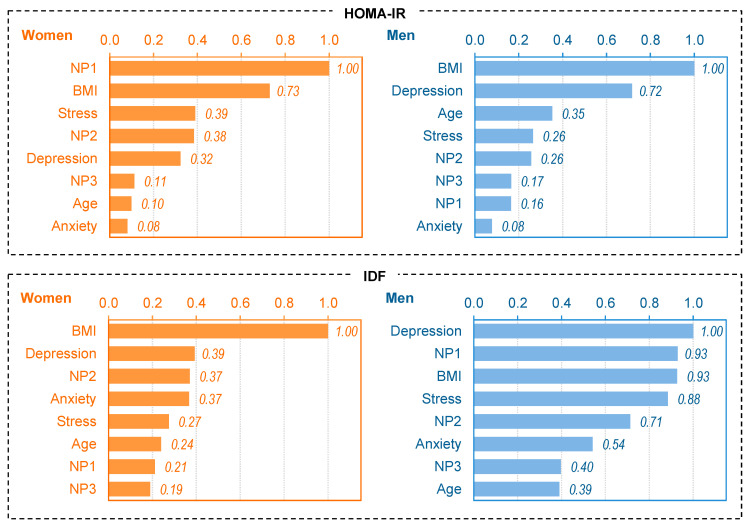
Independent variable importance (normalized values). Note. BMI: body mass index. NP1: minerals and vitamins. NP2: carbohydrates. NP3: Fat and sodium. HOMA-IR: Homeostasis Model Assessment Insulin Resistance. IDF: International Diabetes Federation.

Figure 1 shows the bar charts with the normalized (standardized) scores measuring the importance of the input layer in determining the neural network. All the predictors are represented with a percentage value, resulting in a sensitivity analysis of the relevance of each predictor for the binary classification into MUO/MHO.

For the female subsample considering the HOMA-IR criterion, the model was built for one hidden layer containing three units (excluding the bias unit). The presence of MUO is strongly related with units 3 and 1 in the hidden layer, and different factors seem to increase the likelihood of this unhealthy metabolic condition: (a) older age and low scores for NP1 (mineral and vitamins) and NP2 (carbohydrates) plus higher scores for NP3 (fats and sodium); (b) a psychological state characterized by higher scores for depression and stress. In this model, a protective profile (associated with a greater likelihood of MHO) was characterized by low BMI and depression plus high scores for NP1 (minerals and vitamins). The predictor with the highest (normalized) relevance in this neural model was NP1 (minerals and vitamins), followed by BMI (Figure 1).

**Figure 2:**
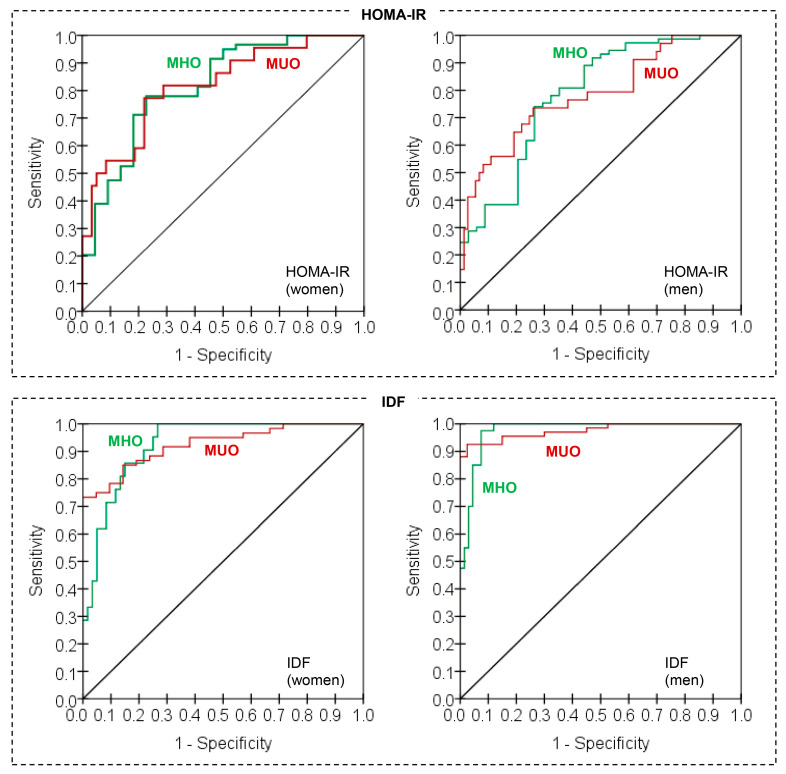
ROC curves obtained in the neural predictive models. Note. HOMA-IR: Homeostasis Model Assessment Insulin Resistance. IDF: International Diabetes Federation. MHO: metabolic healthy obesity. MUO: metabolic unhealthy obesity.

Discriminative capacity was good for both datasets, with overall correct classification rates of 80.9% for the training set and 84.6% for the testing set (Table 3). ROC curves are shown in Figure 2 (see first panel): the area under the curve was equal to 0.817 (95% confidence interval [95%CI]: 0.711 to 0.923).

### 3.3. Neural Network Model for Men Predicting the Presence of MUO (HOMA-IR Criterion)

For the male subsample considering the HOMA-IR criterion, the model was built for one hidden layer containing six units (excluding the bias unit) (Table 2). The profiles that related most strongly to the presence of MUO were: (a) high BMI and high depression and anxiety, and high scores for NP1 (vitamins and minerals); and (b) a psychological state characterized by high depression and anxiety. Two protective profiles (associated with MHO) also emerged: (a) older age plus low BMI; and (b) low BMI plus low scores for NP3 (fats and sodium) and depression. The predictor with the highest (normalized) relevance in this MLP model was BMI, followed by depression (Figure 1).

Discriminative capacity was good for both datasets (overall correct classification rates of around 78% for the training set and 85% for the testing set, see Table 3). The AUC for the ROC curves obtained using the pseudo-probabilities created by the MLP model (Figure 2) was 0.785 (95%CI: 0.688 to 0.881).

### 3.4. Neural Network Model for Women Predicting the Presence of MUO (IDF Criterion)

For the female subsample considering the IDF criterion, the model was built for one hidden layer containing four units (excluding the bias unit). The presence of MUO was particularly high for participants with two profiles: (a) high BMI associated with high depression or anxiety; and (b) high BMI in older age participants with high depression and stress. The likelihood of MHO was higher for participants with low BMI and low stress levels. The predictor with the highest (normalized) relevance in this MLP model was BMI (Figure 1).

Discriminative capacity was good for both datasets (overall correct classification rate of 79.4% for the training set and 100% for the testing set, see Table 3). The AUC for the ROC curves obtained using the pseudo-probabilities created by the MLP model (Figure 2) was 0.925 (95%CI: 0.869 to 0.980).

### 3.5. Neural Network Model for Men Predicting the Presence of MUO (IDF Criterion)

For the male subsample considering the IDF criterion, the model was also built for one hidden layer containing four units (excluding the bias unit). The presence of MUO was mainly related to the following profiles: (a) high BMI plus high stress and low scores for NP1 (minerals and vitamins) and NP2 (carbohydrates); and (b) high BMI in older participants. Low depression was also a strong protector increasing the likelihood of MHO. The predictor with the highest (normalized) relevance in this neural model was depression, followed by scores for NP1 (minerals and vitamins) and BMI (Figure 1).

Discriminative capacity was very good for both datasets (overall correct classification rate of 93% for the training set and 90.5% for the testing set, see Table 3). The AUC for the ROC curves obtained using the pseudo-probabilities created by the MLP model (Figure 2) was 0.975 (95%CI: 0.951 to 1.000).

### 3.6. Assessmen of the Validity of the Neural Predictive Models for Discriminating MUO/MHO

**Figure 3:**
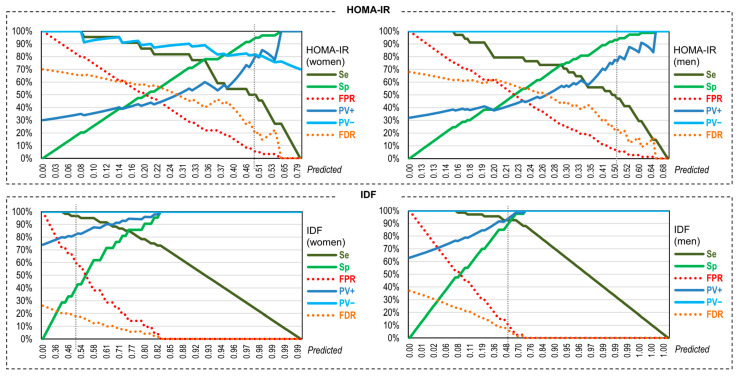
Assessment of the capacity of the neural predictive models. Note. The x-axis refers to the predictions generated by the neural models (for 1 = MUO positive versus 0 = MUO negative). The Y-axis refers to the values of the accuracy indexes (Se, Sp, FPR, FDR, PV+ and PV−) in percentage. Dash line in each graph is plotted in he predicted pseudo-probability 0.50. HOMA-IR: Homeostasis Model Assessment Insulin Resistance. IDF: International Diabetes Federation. Sp: specificity; Se: sensitivity. PV−: predictive value for a negative score. PV+: predictive value for a positive score. FPR: false positive rate. FDR: false discovery rate.

Table 4 shows the results assessing the discriminative capacity of the binary predictions generated by the neural models (1 = MUO positive versus 0 = MUO negative) versus the observed metabolic status (1 = MUO versus 0 = MHO). Figure 3 contains the plot of these validity indexes for the pseudo-probabilities of the neural models for identifying MUO (ranging from 0 to 1). The models obtained for predicting metabolic state based on the HOMA-IR criterion yielded very high Sp and low Se: the models had a high capacity to identify MHO but accuracy at identifying MUO was low. The model created to predict metabolic state based on the IDF criterion demonstrated excellent discriminative capacity for men (Sp and Se around 92.5%), while the model for women also demonstrated excellent Se but very low Sp. Predictive values were between 73% to 95%, with the highest values obtained when considering the IDF criterion. The false predictive rate was low (between 5.5% to 7.5%), except for the model for women considering the IDF criterion (57.14%). The false discovery rate ranged from 11.9% to 20.7%. Kappa values ranged from good to excellent (between 0.47 and 0.84).

## 4. Discussion

The purpose of this study was to evaluate the predictive capacity of nutritional patterns and psychological status in identifying the presence of MUO in young adults with overweight or obesity using neural networks. The results highlight significant differences between men and women in terms of both predictors of MUO and the models’ ability to discriminate between metabolically healthy and unhealthy obesity.

For interpreting the results of this study, it is important to bear in mind that the primary objective was to develop predictive models based on neural networks architecture, as well as to identify the most relevant variables for distinguishing the metabolic health status (MUO versus MHO) among young adults with overweight or obesity. The aim was not to characterize the specific profiles associated with each of the clinical conditions (numerous previous studies have already described the endophenotypes of MUO and MHO individuals, as well as the differences between the HOMA-IR and the IDF criteria) but rather to provide novel predictive tools and assess the potential differences according to the individuals’ sex and the classification criteria employed for defining the metabolic state.

Among the key findings, the predictive model for women based on the HOMA-IR criterion identified that the most relevant factors for MUO were older age, a nutritional pattern characterized by low intake of minerals and vitamins (NP1) and carbohydrates (NP2), along with higher intake of fats and sodium (NP3), and a psychological profile marked by high levels of depression and stress. In contrast, the model based on the IDF criterion found that a high BMI combined with high levels of depression or anxiety increased the likelihood of MUO. Our results are consistent with previous studies indicating that MUO in young adult women can be influenced by several interrelated factors, such as older age contributing to the loss of muscle mass and hormonal changes that promote abdominal fat distribution, which are associated with a higher risk of metabolic disorders [28,59]. Despite the young age of all the women in our study, this factor was identified as having strong predictive capacity. Previous studies confirm our analyses by noting that diets low in micronutrients such as magnesium and vitamin D and an excess of saturated fats and sodium contribute to chronic inflammation, insulin resistance and other metabolic disorders, increasing the risk of MUO [63,64,65,66,67,68]. Previous research has also posited that chronic stress and depression affect the regulation of appetite and physical activity, promoting the consumption of high-calorie foods, and altering the production of hormones such as cortisol, which favours the storage of abdominal fat [69,70,71]. Thus, the interaction between these biological, nutritional, and psychological factors amplifies the risk of developing MUO, highlighting the complex interplay between diet, metabolic health, and mental well-being in young adult women.

For men, the model based on the HOMA-IR criterion showed that BMI, depression, and anxiety were the most relevant factors, also highlighting a protective profile associated with a lower BMI and low levels of depression. The model based on the IDF criterion presented a similar relationship between high BMI, stress, and low scores for NP1 and NP2 with MUO. Our findings confirm previous studies that have suggested that MUO among young adult men is strongly influenced by factors such as a high BMI, depression, anxiety, stress, and insufficient intake of micronutrients and carbohydrates. Specifically, it has been observed that high BMI is associated with a higher amount of visceral fat, which increases the risk of insulin resistance and other metabolic disorders [72,73]. Depression and chronic stress also disrupt the regulation of hormones such as cortisol, promoting abdominal fat storage and chronic inflammation, which are key factors for developing MUO [42,74]. Furthermore, mineral and vitamin deficiencies, along with low carbohydrate consumption, contribute to metabolic dysfunction, while a low BMI and better mental health are associated with a healthier metabolic profile [75]. Together, these factors contribute to a negative feedback loop that increases the risk of MUO in young men [26,76,77,78].

In terms of validation, our findings indicate that models built using the HOMA-IR criterion achieved high specificity but low sensitivity, implying that they were good at identifying MHO, but less effective at identifying MUO. In contrast, models based on the IDF criterion presented better overall discriminatory ability, particularly in the male subsample, with a notable balance between sensitivity and specificity. However, for women, the IDF-based model presented low specificity, suggesting that although it could accurately identify MUO, it was less effective at correctly classifying MHO. Although the application of neural networks to predict MUO is new, such approaches have been successfully used in previous studies for predicting diabetes [79]. However, previous studies have also highlighted limitations, such as low sensitivity in identifying MUO, which is consistent with our findings, especially with the HOMA-IR-based model, which showed high specificity but low sensitivity. Other studies have applied machine learning techniques to analyse aetiology of obesity [80], but they did not test model accuracy based on gender and the criteria used to categorize metabolic and cardiovascular health. Our study provides new empirical evidence on the accuracy of predictive models based on neural networks that differentiate between the classification method used and sex-specific outcomes. The role of physiological and psychological predictors of MUO also vary depending on these interaction factors. The HOMA-IR model is more effective at predicting MUO among men (probably due to the role of visceral fat), while the IDF model, despite being more accurate in men (AUC around 0.98), presents specificity challenges in women. These findings reinforce the need to develop new personalized prevention and intervention plans that account for sex-related differences and the specific metabolic and psychological mechanisms involved in MUO, as has also been suggested by other studies carried out among young age individuals [81].

### 4.1. Limitations and Proposals for Future Studies

This study has several limitations that must be considered when interpreting its findings. As the research specifically targeted a young adult population, the findings may not be generalizable to broader or more diverse populations, or to other life stages, such as adolescence and old age, when metabolic and psychological characteristics vary. Furthermore, although HOMA-IR and IDF are widely used indicators, reliance on these two criteria alone limits the study’s ability to capture all aspects of MUO, such as inflammation or endothelial function. Additional factors that could have influenced the results but were not considered here include physical activity, alcohol consumption, a family history of metabolic diseases. For instance, it is well known that various sociodemographic variables influence the cardiometabolic health of young adults with obesity, and that these contributions may vary depending on geographic and cultural contexts [82,83]. Among the most consistently documented factors in the scientific literature that are associated with increased cardiometabolic risk in this population are sex (men tend to exhibit more impaired lipid profiles, higher blood pressure, and greater visceral fat, while women may have more subcutaneous fat distribution and protective profiles until menopause), educational level (higher education facilitates access to preventive resources and adoption of healthier behaviours), income level (financial problems correlate with less healthy diets and greater chronic stress), and ethnicity (certain minority groups face cultural and structural barriers that hinder access to healthcare).

Future studies are required to assess the consistency of our findings employing other diagnostic criteria to assess metabolic health, such as inflammatory biomarkers. And longitudinal studies are also needed to provide insights on the progression of different obesity profiles over time, including transitions from MHO to MUO.

### 4.2. Strengths

The study has several strengths that significantly enrich the understanding of diverse obesity profiles. These include the multidimensional approach to MUO and the inclusion of a large set of variables measuring nutritional patterns and psychological functioning. Furthermore, this study highlights the specific role of different risk factors of MUO depending on gender, reinforcing the hypothesis that biological and hormonal differences between sexes can influence the manifestation of MUO. In addition, this study represents a novel empirical contribution to the study of the overweight and obesity among young adults, by applying neural network-based models to identify predictors of poor metabolic health. While traditional statistical approaches have been widely used in this field (such as analysis of variance [ANOVA] and regression models), neural networks allow modelling complex, non-linear relationships among large sets of variables that might otherwise remain hidden. To our knowledge, this is one of the first studies employing this statistical approach, offering new insights into early risk factors of MUO and contributing to the advancement of predictive models in metabolic health research.

## 5. Conclusions

The results of this study are consistent with previous studies in identifying BMI, psychological status, and nutritional patterns as key factors for predicting MUO. However, the neural network-based models used in this study appear to outperform those presented in previous research, particularly in their ability to discriminate between MUO versus MHO.

This study provides a more comprehensive view of the multiple factors that contribute to MUO among overweight/obese individuals, as well as the protective factors associated with MHO. The findings can help clinicians to design adequate treatments aimed at achieving metabolic and cardiovascular health among obese patients. The results also have implications for public health policies to promote MUO and early identification of high risk individuals among the general population.

## Figures and Tables

**Table 1:** Descriptive for the sample.

		Total Sample; *N* = 188		Women; *N* = 81		Men; *N* = 107		
		n	%	n	%	n	%	*p*
Marital	Single	148	78.7%	59	72.8%	89	83.2%	0.055
	Married—couple	35	18.6%	21	25.9%	14	13.1%	
	Divorced—separated	5	2.7%	1	1.2%	4	3.7%	
Social	Low	71	37.8%	35	43.2%	36	33.6%	0.163
	Mean	62	33.0%	28	34.6%	34	31.8%	
	High	55	29.3%	18	22.2%	37	34.6%	
		Mean	SD	Mean	SD	Mean	SD	*p*
Age (years-old)		20.76	2.57	20.40	2.62	21.04	2.51	0.090
BMI (kg/m^2^)		28.36	2.94	28.27	2.92	28.44	2.97	0.694
Nutritional pattern 1 (NP1)		7066.55	1883.53	6685.09	1813.72	7355.33	1892.24	0.015
Nutritional pattern 2 (NP2)		271.37	45.46	268.97	46.28	273.18	44.96	0.531
Nutritional pattern 3 (NP3)		77.05	18.36	75.60	16.78	78.15	19.47	0.347
Depression		5.89	1.30	6.12	1.51	5.71	1.08	0.030
Anxiety		5.21	1.55	5.56	1.60	4.95	1.46	0.008
Stress		10.69	2.62	11.11	2.55	10.37	2.64	0.056
Glucose		98.89	11.20	98.40	10.02	99.26	12.04	0.604
Insulin		10.41	2.93	10.39	3.36	10.41	2.57	0.961
Cholesterol-T		193.29	52.11	191.12	44.11	194.94	57.58	0.620
Triacylglycerol (TAG)		147.80	52.43	148.46	52.33	147.30	52.75	0.880
		n	%	n	%	n	%	*p*
Hypertension (HTN)	No	140	74.5%	66	81.5%	74	69.2%	0.055
	Yes	48	25.5%	15	18.5%	33	30.8%	
HOMA-IR criterion	MHO	132	70.2%	59	72.8%	73	68.2%	0.493
	MUO	56	29.8%	22	27.2%	34	31.8%	
IDF criterion	MHO	61	32.4%	21	25.9%	40	37.4%	0.097
	MUO	127	67.6%	60	74.1%	67	62.6%	

Note. MHO: metabolic healthy obesity. MUO: metabolic unhealthy obesity. BMI: body mass index. HOMA-IR: Homeostasis Model Assessment Insulin Resistance. IDF: International Diabetes Federation. NP1: minerals and vitamins. NP2: carbohydrates. NP3: Fat and sodium. Quantitave variables were compared with T-Test procedures, and categorical variables with chi-square tests.

**Table 2:** Parameter estimates of the neural network.

Input Layer	Hidden Layer 1							Output Layer	
HOMA-IR, women	H(1:1)	H(1:2)	H(1:3)				Hidden Layer 1	[MHO = 0]	[MUO = 1]
(Bias)	0.284	0.099	−0.432				(Bias)		
Age (years)	−0.609	0.148	0.151				H(1:1)	0.497	−0.327
BMI (kg/m^2^)	−0.015	−0.550	0.335				H(1:2)	0.322	0.051
NP1	0.598	0.925	−0.357				H(1:3)	0.600	−0.930
NP2	0.641	0.546	0.354						
NP3	−0.455	−0.043	−0.221						
Depression	0.291	−0.795	−0.403						
Anxiety	0.222	0.057	−0.034						
Stress	−0.123	0.054	−0.683						
HOMA-IR, men	H(1:1)	H(1:2)	H(1:3)	H(1:4)	H(1:5)	H(1:6)	Hidden Layer 1	[MHO = 0]	[MUO = 1]
(Bias)	−0.370	0.153	−0.047	0.084	0.129	−0.286	(Bias)	0.386	−0.376
Age (years)	−0.495	−0.257	0.053	0.167	0.119	0.190	H(1:1)	−0.293	−0.080
BMI (kg/m^2^)	0.440	0.719	0.150	−0.385	−0.534	−0.012	H(1:2)	0.079	0.533
NP1	−0.149	0.305	0.073	−0.031	0.055	−0.220	H(1:3)	−0.451	−0.089
NP2	−0.218	−0.056	0.126	0.223	0.139	0.119	H(1:4)	−0.599	−0.645
NP3	0.166	−0.124	−0.501	0.473	−0.344	−0.004	H(1:5)	0.459	−0.273
Depression	−0.022	0.582	−0.006	0.062	−0.459	−0.422	H(1:6)	−0.386	−0.527
Anxiety	−0.433	−0.122	0.039	−0.200	−0.025	0.023			
Stress	0.079	0.588	0.294	−0.331	0.304	−0.453			
IDF, women	H(1:1)	H(1:2)	H(1:3)	H(1:4)			Hidden Layer 1	[MHO = 0]	[MUO = 1]
(Bias)	−0.377	−0.531	1.089	−0.951			(Bias)	0.020	0.818
Age (years)	0.685	−0.244	−0.421	−0.786			H(1:1)	0.119	−0.477
BMI (kg/m^2^)	−0.731	−0.822	2.018	−0.972			H(1:2)	0.376	−0.398
NP1	−0.366	0.640	0.273	0.602			H(1:3)	−0.884	0.671
NP2	0.244	0.192	−0.471	−0.308			H(1:4)	0.211	−0.678
NP3	0.142	0.331	0.263	0.410					
Depression	−0.580	0.051	0.404	−0.593					
Anxiety	−0.100	−0.776	−1.052	0.630					
Stress	0.040	0.445	0.302	−1.362					
IDF, men	H(1:1)	H(1:2)	H(1:3)	H(1:4)			Hidden Layer 1	[MHO = 0]	[MUO = 1]
(Bias)	0.547	−0.728	1.035	−1.076			(Bias)	−1.057	1.345
Age (years)	0.645	0.320	0.855	0.033			H(1:1)	2.112	−2.484
BMI (kg/m^2^)	−1.968	0.264	1.704	−3.141			H(1:2)	0.573	−1.228
NP1	2.429	1.169	0.853	1.782			H(1:3)	−1.229	1.929
NP2	1.054	−0.279	−0.099	0.654			H(1:4)	−3.064	2.220
NP3	−0.035	0.651	0.339	−0.299					
Depression	−0.168	0.544	−0.290	1.985					
Anxiety	0.945	0.557	−0.710	0.599					
Stress	−1.666	0.126	−0.172	0.623					

Note. MHO: metabolic healthy obesity. MUO: metabolic unhealthy obesity. HOMA-IR: Homeostasis Model Assessment Insulin Resistance. IDF: international diabetes federation. NP1: minerals and vitamins. NP2: carbohydrates. NP3: Fat and sodium.

**Table 3:** Classification of the neural predictive models.

		Predicted (Set Training)			Predicted (Set Testing)		
Model	Observed	MHO	MUO	Correct	MHO	MUO	Correct
HOMA-IR	MHO	48	2	96.0%	7	2	77.8%
Women	MUO	11	7	38.9%	0	4	100.0%
	Overall	86.8%	13.2%	80.9%	53.8%	46.2%	84.6%
HOMA-IR	MHO	54	3	94.7%	15	1	93.8%
Men	MUO	16	13	44.8%	2	3	60.0%
	Overall	81.4%	18.6%	77.9%	81.0%	19.0%	85.7%
IDF	MHO	8	12	40.0%	1	0	100.0%
Women	MUO	2	46	95.8%	0	12	100.0%
	Overall	14.7%	85.3%	79.4%	7.7%	92.3%	100.0%
IDF	MHO	29	3	90.6%	8	0	100.0%
Men	MUO	3	51	94.4%	2	11	84.6%
	Overall	37.2%	62.8%	93.0%	47.6%	52.4%	90.5%

Note. MHO: metabolic healthy obesity. MUO: metabolic unhealthy obesity. HOMA-IR: Homeostasis Model Assessment Insulin Resistance. IDF: International Diabetes Federation.

**Table 4:** Assessment of the accuracy of the neural predictive models.

	Sp	Se	PV+	PV−	FPR	FDR	AUC	Kappa
HOMA-IR, women	93.22%	50.00%	73.33%	83.33%	6.78%	16.67%	0.817	0.480
HOMA-IR, men	94.52%	47.06%	80.00%	79.31%	5.48%	20.69%	0.785	0.467
IDF, women	42.86%	96.67%	82.86%	81.82%	57.14%	18.18%	0.925	0.468
IDF, men	92.50%	92.54%	95.38%	88.10%	7.50%	11.90%	0.975	0.842

Note. MHO: metabolic healthy obesity. MUO: metabolic unhealthy obesity. BMI: body mass index. HOMA-IR: Homeostasis Model Assessment Insulin Resistance. IDF: International Diabetes Federation. Sp: specificity; Se: sensitivity. PV−: predictive value for a negative score. PV+: predictive value for a positive score. FPR: false positive rate. FDR: false discovery rate. AUC: area under the ROC curve.

## Data Availability

The raw data supporting the conclusions of this article will be made available by the authors, without undue reservation.

## Supplementary Materials

The following supporting information can be downloaded at: https://www.mdpi.com/article/10.3390/nu17162651/s1. Figure S1: Network diagram (HOMA-IR criterion, women); Figure S2: Network diagram (HOMA-IR criterion, men); Figure S3: Network diagram (IDF criterion, women); Figure S4: Network diagram (IDF criterion, men).
