# Supplementary material for: Predicting Metabolic and Cardiovascular Healthy from Nutritional Patterns and Psychological State Among Overweight and Obese Young Adults: A Neural Network Approach
Source: Nutrients. 2025 Aug 15;17(16):2651. doi: 10.3390/nu17162651 (PMC12389217; doi:10.3390/nu17162651)

Figure S1 (supplementary) *Network diagram (HOMA-IR criterion, women)*

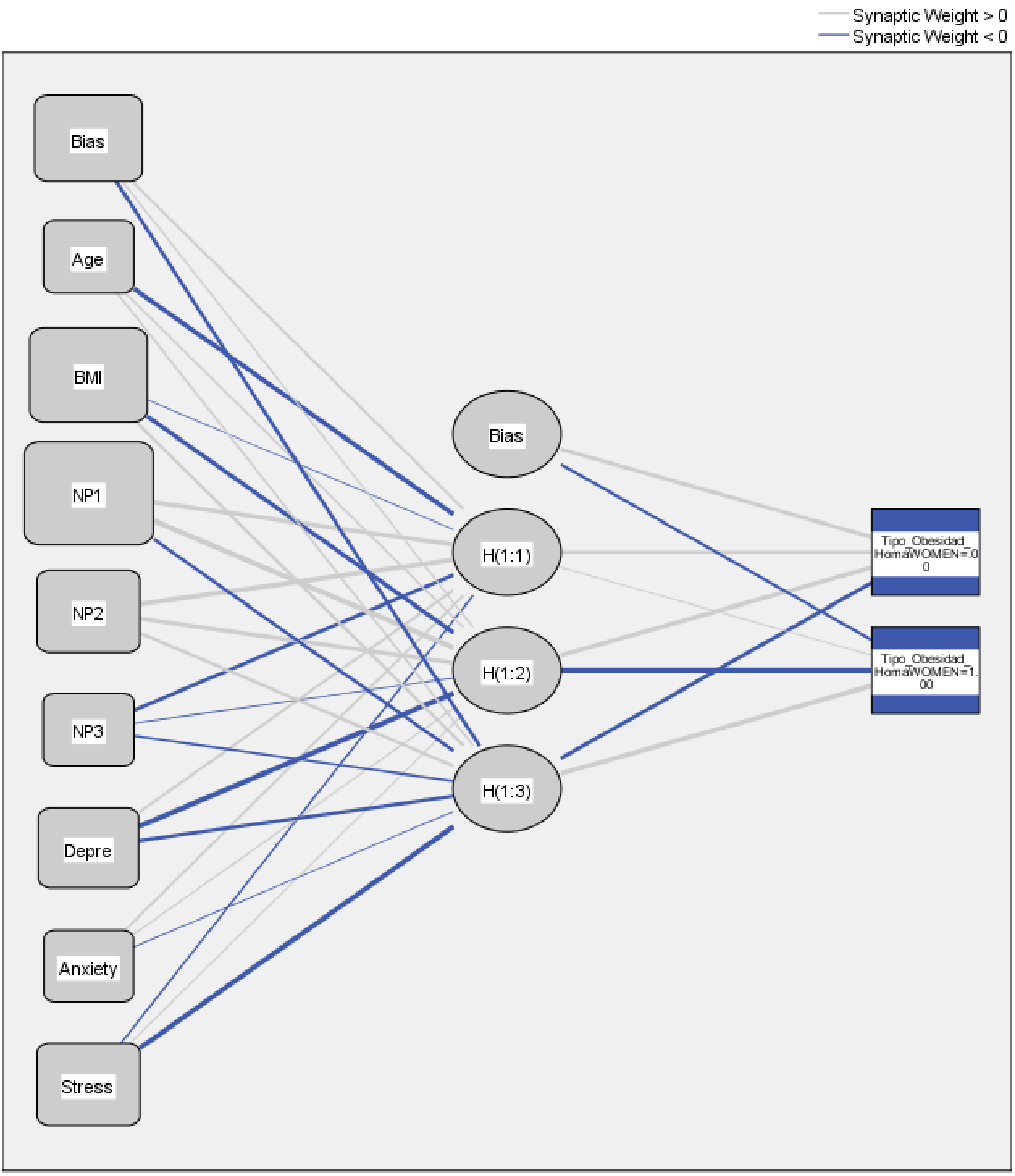

Hidden layer activation function: Hyperbolic tangent

Output layer activation function: Softmax

Figure S2 (supplementary) *Network diagram (HOMA-IR criterion, men)*

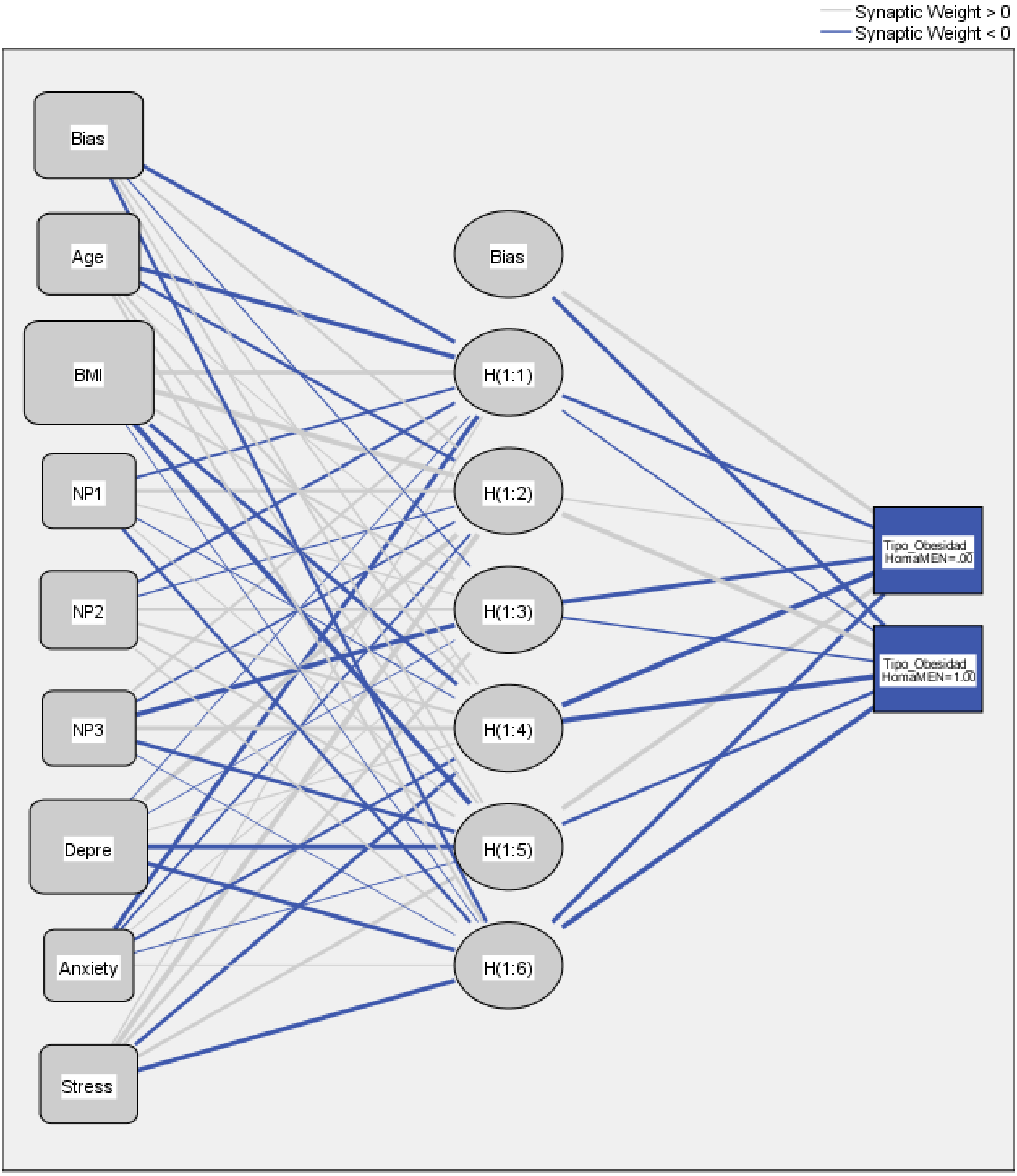

Hidden layer activation function: Hyperbolic tangent

Output layer activation function: Softmax

***Figure S3 (supplementary)** Network diagram (IDF criterion, women)*

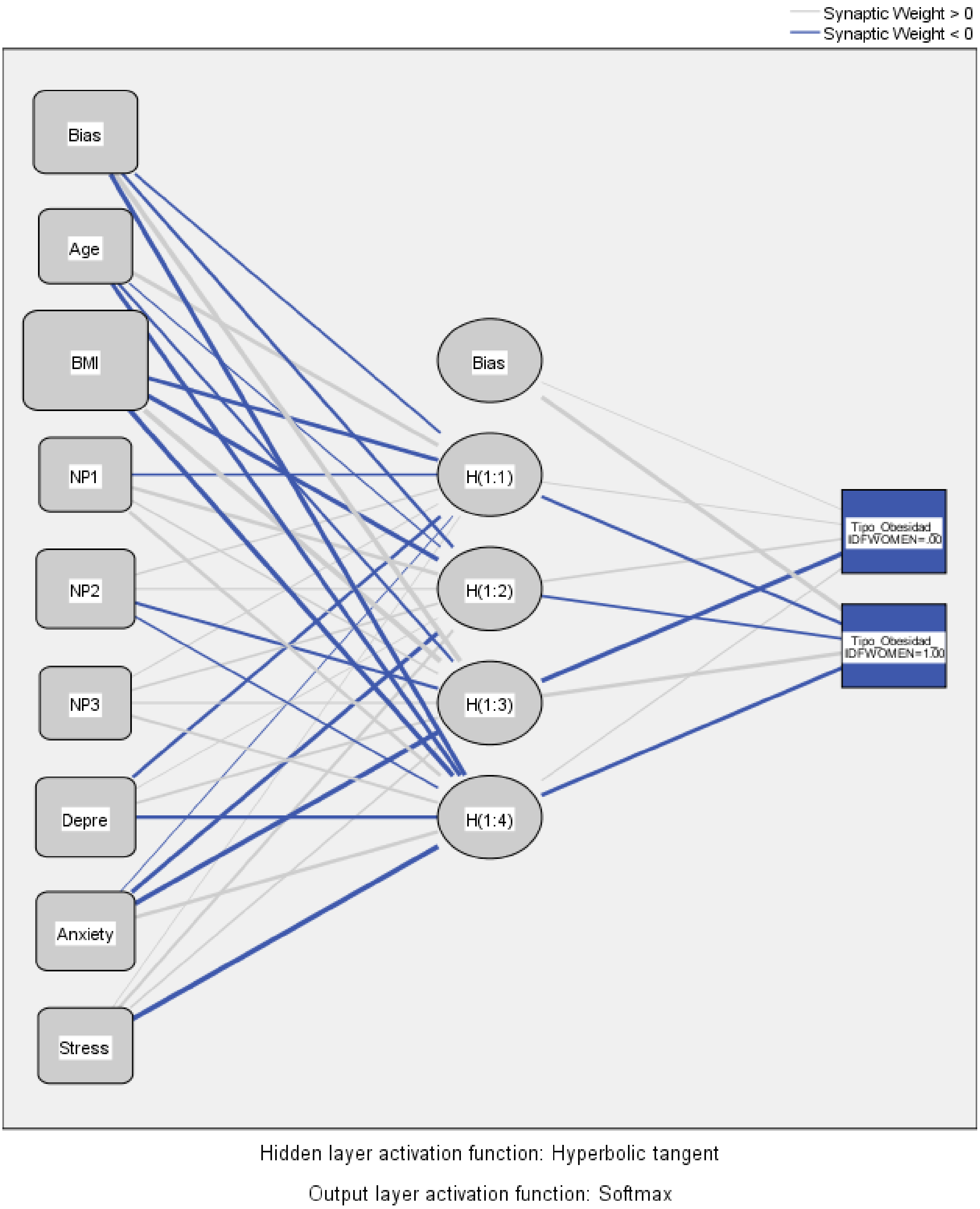

Figure S4 (supplementary) *Network diagram (IDF criterion, men)*

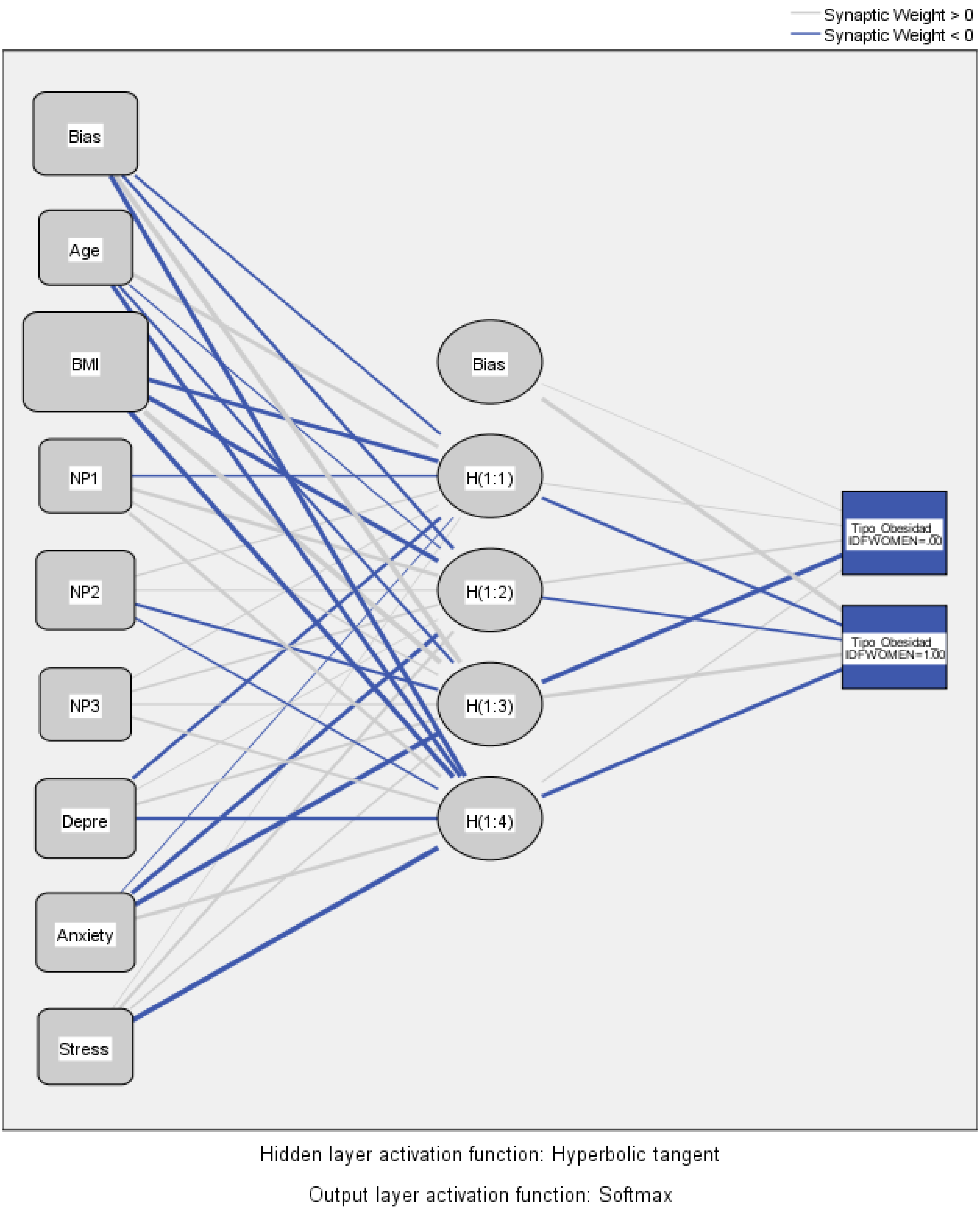

Supplement: Supplementary file 1 [file nutrients-17-02651-s001.zip › nutrients-3767742-supplementary.pdf]
